# Supplementary material for: Are carbon emissions trading and green financial instruments synergistic? -Comprehensive quantitative research based on content analysis
Source: PLoS One. 2024 Mar 7;19(3):e0298601. doi: 10.1371/journal.pone.0298601 (PMC10919601; doi:10.1371/journal.pone.0298601)
Supplement: S1 Data — (ZIP) [file pone.0298601.s001.zip › Data/地级市Z_F工作报告.zip!/░▓╗╒/┴∙░▓╩╨/└┤╫╘╣1⁄2╓┌║┼í╛┬φ┐╦╩2╛▌═°í┐_2019.docx]

各位代表：

现在，我代表市人民政府，向大会报告政府工作，请予审议，并请市政协委员和其他列席人员提出意见。

一、2018年工作回顾

过去的2018年，是本届政府的开局之年。面对错综复杂的外部环境和艰巨繁重的改革发展稳定任务，全市上下以习近平新时代中国特色社会主义思想为指导，全面贯彻党的十九大精神，认真落实中央、省和市委决策部署，坚持稳中求进工作总基调，坚持落实高质量发展要求，以供给侧结构性改革为主线，直面矛盾，正视问题，沉着应对，负重前行，保持了经济持续健康发展和社会大局和谐稳定。

主要做了以下工作：

（一）加快建设现代化产业体系，高质量发展开启新篇章。着力构建“一产向农村特色产业发力，二产向战新产业、先进制造业发力，三产向文化体育旅游、健康医疗养老、现代物流业发力”的现代化产业体系，预计全市地区生产总值1250亿元，增长8%；实现财政收入205.2亿元，增长11.5%。

农村特色产业加快发展。深入实施“五大提升工程”，预计农业总产值增长3.5%。以“一谷一带”“一岭一库”为载体，谋划实施特色产业项目231个，完成投资100多亿元；六安茶谷茶产业综合产值71亿元；淠淮生态经济带特色种养业发展势头良好；江淮果岭林果种植面积45万亩；西山药库中药材种植、加工业年综合产值约20亿元。

工业经济骨骼健康发育。推进“3610”产业体系建设，预计规上工业增加值增幅超过8.5%，六大主导产业增加值占全部规上工业比重达65%；预计技改投资增长10%，实现“机器换人”538台。大力发展战新产业，预计产值增长20%；进入省“三重一创”的“一基地、一工程、一专项”【霍山高端装备基础零部件产业集聚发展基地、金寨先进光伏制造工程、应流小马力航空发动机及特种装备动力应用专项】建设发展态势良好；六安明天氢能燃料电池研发与产业化获批省重大新兴产业专项。常态化开展“四送一服”双千工程，降低实体经济运行成本，减免退企业各类税费33.8亿元。启动实施返乡创业工程，建成农民工返乡创业园12个、小微企业创业基地21个，吸引28万人返乡创办企业1.6万户，“全国支持返乡创业促进脱贫攻坚现场会”在金寨县召开，创新创业的浪潮正在皖西大地上奔流涌动、焕发出勃勃生机。

服务业发展提质增效。开展“放心消费”创建活动，预计实现社会消费品零售总额670亿元、增长12%。推动服务业模式创新和跨界融合，预计实现旅游总收入425亿元、增长21.3%。成功举办第七届大别山（六安）山水文化旅游节暨第四届六安茶谷开茶节；创建1个国家级、5个省级中医药健康旅游基地；悠然蓝溪景区入选国家文化和旅游部《2018文化产业项目手册》；完成65个国家级乡村旅游扶贫村“八个一”工程建设。全年实现农产品上行77.1亿元、增长48%。新增电子商务主体1540个，裕安区获2018年国家电子商务进农村综合示范县，金寨大别山农产品物流园、霍山大别山电商产业园获批省级现代服务业集聚区。

（二）强力推进创新驱动，动能转换迈出新步伐。着力构建以企业为主体、市场为主导、产学研深度融合的创新体系，海龟科技团队成功运营六安大学科技园，新增院士工作站2家、省级备案众创空间5家、工程研究中心1家、工程实验室1家、科技孵化器1家；应流集团跻身首批省级技术创新中心；霍山县“增材制造”成为我市首个入选国家重点研发计划的项目。预计高新技术产业增加值增长15%；裕安经济开发区成功创建省级高新技术产业园区。推进农业科技创新服务平台建设，新认定国家级星创天地3家、省级科技特派员工作站5家。强化知识产权运用和保护，预计新增发明专利授权量260件、增长31%。

（三）持续扩大有效投资，积蓄发展后劲获得新进展。积极开展招商引资，预计到位内资541亿元、增长10%，直接利用外资4.9亿美元、增长12%；新引进亿元以上项目241个、5亿元以上项目46个。产业基金撬动投资新模式破题开局，主投跟投市内产业项目8个。强化政银担企对接，全年新增贷款309亿元；开展“千名行长进万企”活动，缓解融资难，仅小微企业就增加融资16亿元；培育优质上市挂牌后备资源，直接融资62亿元。运用PPP模式，引导社会投资97亿元。落实“四督四保”和“五项机制”，预计固定资产投资增长15%，新开工亿元以上重点项目200个、完成投资897亿元，中科瑞华产业园、迎驾野岭产业园、第6代显示模组生产基地、明天氢能产业园、顺怡隆智能制造项目、绿色板材工业园、美盈森等一批重大项目建成或加快推进。300万吨钢企重组成功，165万霍邱人民梦圆钢城指日可待。

（四）扎实打好三大攻坚战，年度战役赢得新胜利。脱贫攻坚战再战再捷。全年承办两次全国扶贫工作现场会，精心打造扶贫项目库、光伏扶贫等“六安亮点”，金寨县荣获全国脱贫攻坚组织创新奖。积极推进“四带一自”产业扶贫、“三有一网”点位扶贫、“三业一岗”就业扶贫，新增扶贫小额贷款11.7亿元，完成贫困户危房改造1.9万户和易地扶贫搬迁1.15万人，全面落实教育和健康脱贫政策。聚焦深度贫困地区，投入4.3亿元改善行蓄洪区生产生活条件。积极利用城乡集体建设用地增减挂钩政策，加快行蓄洪区土地和村庄整治。预计15.1万贫困人口脱贫、181个贫困村出列，舒城、金安、裕安3个县区有望卸掉贫困县的“紧箍咒”，开启追梦小康新航程。

生态环境质量持续改善。组织开展“三清零、七聚焦、六强化、一提升”专项行动，狠抓环保督察检查发现问题整改。PM_2.5_年平均浓度创近年来最低，完成省政府年度考核目标。地表水断面及饮用水水源国考监测100%达标。深入开展“绿盾2018”自然保护区专项行动，81个问题全部整改到位。完成9条城区黑臭水体和116个入河排污口整治。秸秆综合利用率达91%。化肥、农药使用量实现负增长。水资源和能源“双控”年度考核居全省优秀行列。在中央环保督察“回头看”中，全市干部群众用“不被问责一人”的底气，展现了老区人坚韧务实、勇于胜利的豪气。

防范化解重大风险成效显著。严厉打击非法集资活动，稳妥化解涉众型经济案件风险。支持地方法人金融机构化解风险，积极开展互联网金融风险专项整治、P2P网络借贷风险防控和非法交易场所清理整顿。完成政府隐性债务清理甄别，严格执行政府债务限额管理制度。切实加大对社会稳定、安全生产、网络舆情等领域敏感性、潜在性风险的预警研判，谋实应急预案，注重源头化解，加强舆情监控，严防重大负面事件再次发生。

（五）积极实施乡村振兴战略，农业农村发展呈现新气象。实施全市乡村振兴五年规划。加强耕地保护，划定粮食生产功能区和重要农产品保护区。全年粮食总产69.2亿斤，新增“三品一标”认证产品53个，地理标志证明商标数全省第一。成功举办首届中国农民丰收节。实施农村人居环境整治行动，推进农村垃圾、污水、厕所专项整治“三大革命”，全面完成农村陈年垃圾清理，启动51个乡镇污水处理设施建设，改厕5.9万户。加强乡风文明建设，创建县级以上文明村镇579个、移风易俗乡村示范点30个。实施农村土地整治行动，新建高标准农田32.3万亩。全力推进农村公路建设，完成农村道路畅通工程2660公里、“四好农村路”扩面延伸工程2304公里，舒城县荣获全国“四好农村路”建设示范县。实施农田水利“最后一公里”建设，治理面积79.1万亩。推进农村饮水安全工程，解决90万人饮水安全问题。农村土地“三权分置”改革取得重要进展，农村产权交易市场逐步建立。完成农村“三变”改革首批试点和37.7%建制村的集体经营性资产股份合作制改革任务。

（六）进一步深化改革开放，激发发展活力取得新成效。扎实推进农村金融改革等36项国家级改革试点和涉农资金整合改革等26项省级改革试点。加大“放管服”改革力度，完成“证照分离”改革试点任务，“一网一门一次”改革取得突破，“减证便民”市级申请材料精简56.2%。扎实推进商事制度改革，新增企业增长12.2%。顺利完成国税地税征管体制改革。深化财政体制改革，健全市与叶集区财政管理体制。平稳推进盐业体制改革。推深做实河长制、湖长制、林长制，建成县乡村三级公路路长制管理体系。积极做好市县机构改革准备工作。

对外开放不断扩大。大力拓展对外贸易，积极落实扶持政策，预计实现进出口8.4亿美元、增长16%；组织近30家企业亮相世界制造业大会和中国国际徽商大会，200家企业参加首届中国国际进口博览会，23家次企业参加8个境外展，27家次企业参加11场对外贸易交流活动。

（七）着力推进城乡协调发展，城镇建设管理打开新局面。启动城市总体规划修改，审查批准了三十铺镇、城南镇等10个乡镇建设规划，强化规划管理，硬化规划执行。积极创建全国文明城市、国家卫生城市，成功获得全国文明城市提名。完成市本级政府性投资86.4亿元，火车站站房和广场改扩建、佛子岭中路改造等项目建成。启动城市“双修”专项行动。以全面解决历史遗留问题为突破口，重塑征迁安置工作机制，实现征迁安置同步同向。一批住房困难群众“出棚进楼”。开展“安置小区双清”专项行动，加快58个历史安置小区清产清账；启动“两补一化”专项行动，对23个安置小区实行补差补缺和管理规范化；开展 “问题楼盘”专项治理，汇金国际等22个“问题楼盘”化解取得重要进展；积极开展“历史遗留毛地出让地块”专项整治。新建177万平方米10个安置小区；开展“让征迁市民早日安家”专项行动，让1.2万户3.5万人结束长期过渡安置生活，实现“回家过年”的愿望。

县城及镇村建设加快推进。推动完善县城总体规划、控制性规划和多规合一，规划水平不断提高。县城建设持续发力，“两治三改”、黑臭水体治理、地下综合管廊建设等加快推进，全年完成政府性投资103亿元，功能配套不断完善。统筹特色小镇、历史文化名镇、传统村落建设，裕安区苏埠镇入选中国历史文化名镇，金寨电商小镇、霍山石斛小镇获批省级特色小镇，霍邱县临水镇、金寨县长岭乡、舒城县汤池镇获批安徽省千年古镇，毛坦厂镇浸堰村、天堂寨镇前畈村入选中国传统村落名录。持续抓好美丽乡村建设，整治建设47个乡镇政府驻地、221个省级中心村、180个县级中心村。

基础设施建设不断加快。完成交通投资71.9亿元，新建、续建国省干线公路642公里，山湖旅游大道建成通车，S366合六南通道（六安段）、G312六安西至大顾店段开工。合安高铁线舒城东站开工建设。完成水利建设投资14.4亿元，淮干临王段加固工程基本完成，淠河治理工程主体完工，史河治理工程竣工，6条中小河流治理项目和82座小水库除险加固项目主体工程完工。完成电网建设投资17.4亿元，新建、续建大中型电网工程20项。

（八）大力保障和改善民生，人民幸福感得到新提升。预计城镇、农村常住居民人均可支配收入分别增长8%以上和9%以上。投入财政资金110亿元，完成33项民生工程。实施就业优先政策，城镇新增就业6.53万人。城乡居民基本养老金标准提高50%。城乡低保标准及最低工资标准提标升档。启动城市社区康养中心建设和农村失能特困人员集中养护试点。新建改扩建公办幼儿园31所；完善城区学校建设布点规划，新建改扩建中小学6所；深入实施高中教育普及攻坚计划，完成项目51个；建设“全面改薄”校舍项目3112个。医院现代管理、基本医疗卫生和医疗保障制度不断完善，县域医共体实现全覆盖，乡村医生“六制”管理落地见效。成功举办淠史杭工程开工建设60周年纪念活动，广泛开展“社区一家亲·文化进万家”和“美丽乡村行·文化乐万家”系列文化品牌活动，新建国家贫困县村级综合文化服务中心47个；成功举办六安国际马拉松赛、舒城首届国际自行车赛、省第二届健身休闲大会，在省第十四届运动会、第八届少数民族运动会上金牌数、奖牌总数和团体总分均创新高。市区和金寨县城乡公交一体化成功运营，252万群众踏上了进城的“直通车”。

社会治理能力进一步提升。深入开展“深重促”专项行动，大批合理信访诉求得到解决，不合理信访诉求受到教育，违规信访行为受到约束，实现进京和赴省上访“双下降”、信访公信力和满意度“双提升”。扎实推进扫黑除恶专项斗争，共破案315起，群众安全感、满意度稳步上升。做实“铸安”行动，安全生产形势企稳向好。强化食品药品安全监管，成功创建省食品药品安全城市。加强综合防灾减灾救灾体系建设，应急处理能力不断提高。推进“七五”普法，加强法律援助工作。启动第四次全国经济普查工作。巩固双拥模范城（县）成果，奋力推进国防动员和后备力量建设发展，军政军民更加团结。广播电视、新闻出版、人防、地震、档案、地方志、民族宗教、外事侨务、对台事务、气象、工会、共青团、工商联、科协、妇女儿童、老龄、残疾人等各项事业取得新进展。

（九）切实加强政府自身建设，运行效率实现新提高。坚持把政治建设摆在首位，深入推进“两学一做”学习教育常态化制度化，扎实开展“讲严立”专题警示教育，全力抓好省委巡视反馈问题整改，严格落实意识形态工作责任制。深刻总结经验教训，着力强化政治纪律和政治规矩，着力改变自由散漫和不作为乱作为现象。建立市政府领导重点工作清单管理制度，月月对账销号。理顺体制机制，加强勤政建设，政府系统运行效率明显提高。落实重大事项合法性审查、行政复议和行政应诉制度，规范行政执法行为，扩大政务公开，法治政府建设进一步加强。自觉接受人大监督，依法执行人大决议决定，办理人大议案2件、代表建议156件。主动接受政协民主监督，办理政协委员提案253件。锲而不舍纠正“四风”，推进审计监督全覆盖，着力营造风清气正的良好政治生态。

各位代表：

刚刚过去的2018年，全市广大干部群众用辛勤汗水换来了数不胜数的成绩，彰显了一分耕耘一分收获的真理力量，坚定了“为有牺牲多壮志，敢教日月换新天”的信心决心。在此，我谨代表市人民政府，向奋斗在各条战线的全市干部群众表示崇高敬意和衷心感谢！

各位代表：

在我们前进的道路上，还存在不少困难和问题：发展不充分不平衡问题突出，经济总量不大、人均水平较低困局未解；建设现代化经济体系工作十分紧迫，创新驱动发展实体经济、民营经济任务十分艰巨；贯彻新发展理念、转变发展方式任重道远；教育、就业、医疗、养老等民生领域诸多短板十分明显；全省脱贫攻坚决胜之役在我市深山区和行蓄洪区。思想观念赶不上趟、担当作为挂不上档的现象依然存在；会议多、文件多、考核多的现象还比较严重；“四风”问题禁而未绝，违纪违法现象还时有发生。对此，我们要进一步增强忧患意识和使命担当，坚持民之所盼就是政之所向，下大力气解决这些问题，决不辜负六安人民的信任和重托！

二、2019年工作安排

各位代表：

2019年是新中国成立70周年，是全面建成小康社会关键之年。做好今年各项工作，意义重大、影响深远。

2019年全市经济社会发展的总体要求：以习近平新时代中国特色社会主义思想为指导，全面贯彻党的十九大和中央、省委经济工作会议精神，按照市委四届八次全会总体部署，坚持稳中求进工作总基调，坚持新发展理念，坚持高质量发展要求，坚持以供给侧结构性改革为主线，坚持深化市场化改革、扩大高水平开放，统筹推进稳增长、促改革、调结构、惠民生、防风险工作，进一步落实“六稳”措施，坚定不移把发展作为第一要务，坚定不移以经济建设为中心，坚定不移打好三大攻坚战，坚定不移增强人民群众获得感、幸福感、安全感，保持经济社会持续健康发展和社会大局稳定，以老区改革发展稳定优异成绩庆祝中华人民共和国成立70周年！

2019年经济社会发展的主要预期：全市地区生产总值增长8%，财政收入增长9%，规模以上工业增加值增长8.5%，固定资产投资增长12%，社会消费品零售总额增长11%，城镇常住居民人均可支配收入增长8.5%，农村常住居民人均可支配收入增长9%，城镇登记失业率控制在4.5%以内，完成省下达的生态环境年度考核目标。

2019年经济社会发展重点任务：

（一）持续深化供给侧结构性改革，加快高质量发展步伐。坚持高质量发展要求不动摇，加快现代化产业体系建设，努力实现质量变革、效率变革、动力变革。

突出发展农村特色产业。坚持保护特色、发掘特色、塑造特色、发展特色。提升“一谷一带”“一岭一库”发展平台，打造六安瓜片、霍山石斛、皖西白鹅、六安林果、霍寿黑猪、万佛湖鱼头等特色产业链条；扩大小龙虾产能，推动小龙虾在地餐食和在地加工、冷链运输冰鲜上市。推进适度规模经营，提高规模效应。推进主体经营，发展农业企业、农民合作社和家庭农场。推进一二三产融合经营，拓展农业功能，大力发展休闲农业和乡村旅游。推进电子商务进农村综合示范建设。实施特色品牌发展战略，新增一批“三品一标”认证产品。整合农业发展专项资金，重点支持特色产业发展。

突出壮大工业经济骨骼。推进“3610”产业体系建设和工业转型发展“十大行动”。确保应流航空产业园加快建设、明天氢能产业园一期投产、300万吨钢厂项目建成投产；推进六大主导产业集中度达70%以上、装备制造业首位度达25%以上。加快运用互联网、物联网、大数据和人工智能改造提升传统生产方式，力争技改投资增长15%以上、“机器换人”500台以上。加快培育特色产业集群，着力在六安城区汽车零部件、霍邱钢铁、金寨清洁能源、霍山高端装备基础零部件、舒城电子信息、叶集家居制造等产业领域打造超百亿级产业集群。加快推进省级重大新兴产业“一基地、一工程、两专项”和市级战新基地建设。建立按月调度督查制度，推动线索项目早签约、签约项目早落地、落地项目快建设。

突出发展现代服务业。坚持在合肥都市圈中错位布局、互补发展。结合城市经营，着力引导社会力量在我市投资发展文化体育旅游、健康医疗养老、现代物流等服务业项目。加快传化物流园、邮政物流园、富春物流中心、电子商务产业园和城乡配送中心建设，完善生产资料供应链和城乡快递服务网络；积极培育和发展研发设计、信息技术服务、融资租赁等生产性服务业。支持现代保险服务业发展，拓展保险市场的风险保障功能。加快旅游基础设施和服务设施标准化建设，推进大别山主峰创建国家5A级旅游景区。深入推进农贸市场、便民超市改扩新建。

突出推进开发（园）区高质量发展。围绕“工业强市主阵地、高质量发展增长源”的功能定位，按照“三融合三为主”发展思路，加快推进开发（园）区转型升级。完成8个省级以上开发（园）区优化整合。推进总体规划、产业发展规划编审和报批。推进生产要素向工业倾斜、工业项目向开发（园）区集中。推动开发（园）区培育首位产业和主导产业，建设发展科创园。塑造羽毛球、童车童床、婚纱、功能雨衣等特色工业小镇。做大做强龙头企业，培育“专精特新”骨干企业，形成产业集聚和集群。坚持节约集约和依法依规利用土地，严控项目用地规模；开展僵尸企业重组行动，让沉淀资源焕发生机活力。健全开发（园）区发展分类评价体系和调度通报制度，力促各开发（园）区在全省争先进位。

突出增强科技创新能力。加大高新技术企业培育力度，确保增加值增长10%。鼓励和支持企业组建产业技术研究院、院士工作站、博士后工作站、技能大师工作室、劳模（职工）创新工作室等产业创新平台，推动六安高新技术产业园区高标准建设，加快六安大学科技园二期工程、合肥高新区霍邱现代产业园、氢能产业研究中心建设。实施知识产权质量提升工程，力争万人发明专利拥有量达2.6件以上。强化科普能力建设，提升公民科学素质。推进产业工人队伍建设改革，弘扬劳模精神和工匠精神。实施更加有效的人才引进和培育政策，打造人才“强磁场”，广聚英才、用好人才，充分激发各类人才积极性，真正把“人才是发展的第一资源、创新是发展的第一动力”从都会喊的口号变成都会干的行动。

（二）全力打好三大攻坚战，确保实现连战连捷。集中优势兵力，一仗接着一仗打，确保脱贫攻坚任务全面完成，确保生态环境质量持续改善，确保重大风险得到有效防范。

打好精准脱贫攻坚战。坚持“65232111”扶贫精准要求，坚持尽力而为、量力而行，既不降低标准，也不吊高胃口，向绝对贫困发起总攻，确保8万左右贫困人口脱贫、剩余贫困村全部出列、霍邱县和金寨县高质量“摘帽”。全面落实省级加强大别山等革命老区脱贫攻坚各项政策措施，大力实施扶贫重点工程，打造“四带一自”产业扶贫、 “三有一网”点位扶贫、“三业一岗”就业扶贫升级版，推进资产收益扶贫和小额信贷扶贫，全面落实健康扶贫政策，实现建档立卡贫困户危房改造“应改尽改”。采取有效措施防止脱贫人口返贫，研究解决收入水平略高于贫困户的群体缺乏政策支持等新问题。启动实施沿淮行蓄洪区安全建设三年行动计划，加快庄台和保庄圩环境整治、提标扩容。加强对贫困老年人、残疾人、重病患者等群体的精准帮扶。

打好污染防治攻坚战。坚决打好“七大标志性战役”，加快城市黑臭水体治理，推进餐厨剩余物、污水和建筑垃圾等处理设施建设。强化农业面源污染防控与治理，建立土壤环境质量状况定期调查制度。加快生活垃圾焚烧发电项目二期建设，启动病死畜禽集中无害化处理中心建设。创新生态补偿方式，稳步推进地表水环境质量、空气环境质量和重点生态功能区等生态补偿。开展生态保护红线勘界定标，坚决守住自然保护区、森林公园、风景名胜区、湿地公园、地质公园、城镇开发边界等警戒红线。开展生态区域违法建设问题专项整治。推进自然资源资产审计。严格执行排污许可制度。坚持严格执法和加强服务相统一。完成第二次全国污染源普查。健全完善河长制、湖长制。推深做实林长制改革。积极创建全国绿化模范城市。让绿色成为底色，蓝天成为标配，把“绿水青山”的六安品牌擦得更亮！

打好防范化解重大风险攻坚战。切实履行金融监管职责，严厉打击金融诈骗、恶意逃废债、非法集资等违法犯罪活动；支持农村商业银行处置不良资产，提升金融素质，增强发展能力。加强政府债务管理，力争国家给六安多发债券、多筹资金。落实年度债务还本付息计划。建立健全房地产市场监测引导体系，确保房地产市场平稳有序。坚持加强社会稳定、安全生产和网络舆情等领域重大风险隐患排查，坚持抓早抓小、靠前指挥，坚决防止“小事拖大、大事拖炸”的黑天鹅或灰犀牛事件。

（三）加快新型城镇化进程，统筹推进城乡发展。城镇化是现代化的必由之路。要遵循规律、把准方向、因势利导、加快推进，为城乡居民带来更多福祉。

加强中心城市规划建设与管理。规划的遗憾是永久的遗憾。坚持规划先行，完善总规详规和专项规划体系，突出城市特色风貌，强化重点片区城市设计。坚持规划与建设“一张图”，防止规划与建设“两张皮”。深入开展全国文明城市、国家卫生城市、绿色生态试点城市、海绵城市、智慧城市创建行动。推进数据化信息化建设，迎接六安5G时代到来；完善城市路网，推进城市环线畅通工程，加快长安北路和西环路快速化改造，新建19条城区支路。补齐城市功能短板，加快推进城市污水管网、城北污水处理厂二期等工程，新建停车场10个。继续开展城建征迁安置领域六大专项行动，着力解决规划建设、征迁安置遗留问题，加快推进安置房项目建设。建立健全城市管理新体制机制，充分发挥区街社区组织在运维管理城市中的创造性，充分调动广大市民共建共管共享城市文明的积极性。下绣花功夫管理城市，推动城市精细化管理，让广大市民生活得更安心、更舒心、更省心！

加强县城和乡镇建设。持续建设宜居县城，优化各县空间布局，完善县域规划体系，提升县城和乡镇规划水平。加大违法建设、违法用地“两项治理”，推进棚户区、城中村、老旧小区“三大改造”，补齐教育、文化、医疗、养老等公共服务领域短板。统筹考虑乡镇差异化、特色化、品质化发展，以特色小镇、历史文化名镇等为抓手，重点支持基础条件好、发展潜力大、人口集聚多、产业支撑强的重点镇开展示范建设，提升乡镇综合承载能力。

（四）深入实施乡村振兴战略，加快农业农村现代化。乡村振兴是新时代“三农”工作的总抓手，要围绕五年规划，聚焦阶段任务，着力抓重点、补短板、强弱项。

坚持强化规划引领。加强永久基本农田保护，落实市县乡三级规划，优化乡村空间布局，促进生产、生活、生态协调发展。完善城镇和乡村体系规划建设，实现以镇带村、以村促镇，推动现代城镇与田园乡村合理分工、功能互补和融合发展。

坚持推进生态宜居乡村建设。加大力度有序推进集聚提升型、城郊融合型、特色保护型等三类村庄建设和搬迁拆并型村庄土地整治。用足用活土地增减挂钩政策，盘活农村集体建设用地存量，推进集镇、永久村庄规划建设。加强农村房建规划建设管理，消除农村房建无政府状况。全面深化农村环境“三大革命”，推进乡镇政府驻地生活污水处理设施全覆盖，推进农村生活垃圾源头分类和资源化利用试点示范工作，完成农村改厕5万户。高质量推进“四好农村路”建设，实施扩面延伸工程3370公里；实施农村饮水安全巩固提升工程；推动农村电网改造升级。

坚持提升乡村善治水平。践行社会主义核心价值观，繁荣乡村文化，建立完善村规民约，深化文明村镇创建，拓展新时代文明实践中心功能，促进移风易俗。提升现代乡村治理能力，完善县乡村三级综治中心功能和运行机制，扩大农村社区建设试点，深入开展“民主法治示范村（社区）”创建活动。强化新型职业农民、农村实用技能人才、乡村企业家和“三农”工作队伍建设。

坚持深化农村综合改革。深入推进农村集体资产产权制度改革，逐步建立健全农村产权“确权、赋权、管权、活权”体系。扎实推进 “三变”改革，继续推进 “三权分置”，分类建立农村产权交易市场。健全闲置宅基地自愿有偿退出机制，完善土地征收制度，拓展集体经营性建设用地入市范围。

（五）大力支持民营经济，激发发展活力。民营经济地位举足轻重，作用不可替代。坚持“为自己人办事就是办自己的事”的观念，让六安成为民营经济千帆竞发的港湾。

加大支持和保护力度。支持民营企业创新发展，培育一批“专精特新”中小企业。严格落实减税降费系列政策，降低民营企业税费负担。破除各种隐性障碍和歧视性待遇，营造公平竞争环境。强化法治保障，保护民营企业合法合规权益、企业家人身和财产安全。着力构建“亲”“清”新型政商关系。继续实施企业家素质提升工程，进一步激发和弘扬企业家精神。

加大 “四最”营商环境建设力度。持续推进“放管服”改革，优化完善政府权责清单制度。深化商事制度改革，全面推开“证照分离”改革，纵深推进“双随机、一公开”监管。常态化推进“四送一服”双千工程。深化“互联网+政务服务”，推动政务服务“一网一门一次”改革再升级，坚决除烦苛之弊、施公平之策、开便利之门，让企业和群众办事像“网购”一样方便。

加大返乡创业工程推进力度。以开发（园）区为载体，加快建设一批高水平双创平台。加快市级返乡创业示范园认定，完善配套政策体系。力争创建国家级返乡创业类平台2个、省级平台5个，打造10个市级返乡创业示范园，培育一批成长性强的创业示范企业。充分发挥驻外招商联络处、在外人才协会、异地六安商会等桥梁作用，加强政策宣传，大力引导返乡创业。

（六）全面深化改革开放，释放发展动力。惟改革者进，惟开放者强。要通过改革破除阻碍，通过开放强化动能，真正做到“改革不停顿、开放不止步”。

提升重点领域改革效率。完成市县机构改革任务，精准制定改革方案，精细抓好组织实施。加快推进事业单位分类改革、综合执法体制改革。全面实施预算绩效管理，加快构建全方位、全过程、全覆盖的预算绩效管理体系。推动融资平台公司转型发展。深化社会治理领域改革，推进社区治理创新和智慧社区建设。统筹推进司法、审计、环保等行业体制改革。

提升开放合作水平。扩大进出口贸易，推动出口市场多元化。围绕共建“一带一路”，鼓励、支持龙头企业在海外建立生产基地和营销网络。推进海关特殊监管区域建设。积极有效利用外资，加强对外经济交流合作，办好第7届中国国际羽绒博览会和第65届国际羽绒羽毛局年会。加强与友好城市国际合作。着眼长三角一体化发展，着手合肥都市圈一体化建设，在产业发展联动、基础设施互通、金融服务合作、科技成果转化、生态环境联防中加快一体化发展进程，在加快建设合六经济走廊中彰显一体化发展成果。抢抓淮河生态经济带上升为国家战略的重大机遇，制定发展行动计划，与淮河生态经济带城市联动，打造水清岸绿产业优的美丽新淠淮，让淠淮群众远离水患、告别贫困、安居乐业！

（七）着力扩大有效投资，增强发展后劲。今天的投资就是明天的生产力，没有有效的投入就没有有效的产出。要确保新增项目不断档、有效投入不减速，全年完成亿元项目投资900亿元以上。

全力抢抓国家加大投资政策机遇。大力推进沿江高铁合肥至武汉段、合六与合新六城际铁路、金寨支线机场、金安通用机场、合肥-霍邱-阜阳高速前期工作。完善国省干线公路建养资金投入机制，新建、续建国省干线公路328公里。推进重点水利工程建设，完成淮干一般堤防加固，开工建设杭埠河（丰乐河）综合治理工程，推进6条中小河流治理和110座小水库除险加固项目，加快实施淠河总干渠（九里沟—青龙堰）东部新城水利综合治理工程。推进霍山抽水蓄能电站项目可研工作。新建、续建21项大中型电网工程。加快合六天然气联络线、金安至叶集至金寨天然气支线工程建设，实现六安城区双气源保障。

全力激发社会领域投资活力。坚持“非禁即入”，认真实施市场准入负面清单制度。发挥财政性资金带动作用，通过基金风投、股权投资等多种方式，引导各类社会资本加大对主导产业和重点项目的投入。完善政府和社会资本合作模式，鼓励社会资本参与政府出资的融资担保公司。优化投资环境，让更多社会资本在六安着床！

全力拓宽金融投资渠道。积极畅通金融血脉，优化金融服务监管和激励考核，加大对重大项目、基础设施、科技创新、“三农”和小微企业融资支持，发展普惠金融，继续开展“千名行长进万企”活动，着力解决企业融资难、融资贵问题。深入发掘和培育优质上市挂牌后备资源。积极利用多层次资本市场，力争实现直接融资56亿元。

（八）加大招商引资力度，提升质量和效益。优化招商引资工作思路。坚持算大账不算小账、算长远账不算眼前账，反对不算账的亏本买卖。推动由不计代价到讲求质效、由政策粗放到举措精细、由等商上门到主动出击、由领导幕后指挥到亲临前线出征的转变。力争全年实现到位内资595亿元、增长10%，直接利用外资5.2亿美元、增长7%。

积极推进产业招商。围绕主导产业，深化研究，细化内涵，形成产业链企业库，按图索骥。瞄准全国领先行业、前位企业主动出击，大力引进产业层次高、集聚能力强、带动效应大、延伸配套好的支柱型项目。锁定相关配套企业，壮大、补齐、拓展产业链条，促进产业集聚集群发展。

积极创新招商引资方式。推动政府招商与市场化招商相结合，加强与金融、投资机构的深度合作，积极运用产业投资基金、股权投资基金等产融结合的资本招商新模式。广泛对接专业咨询机构、行业协会、商会等，有针对性开展委托招商。开展专题招商，推进以商招商。

积极完善招商引资流程。按照经济、简约、易行原则，优化招商引资优惠政策。落实落细国家、省扶持产业的系列政策，注重政策协同，促进项目落地。掌握国家产业政策、生态环保限制性规定，健全招商引资项目论证机制。做实环境影响评价、水资源评价、节能评价等前期工作，强化项目管理服务，确保签约项目快速落地、落地项目快速开工。严格招商合同管理，坚持重诺守信，强化双方履约跟踪督导。

（九）推动社会事业全面进步，不断增进民生福祉。坚持以人民为中心的发展思想，高质量推进33项民生工程，织密扎牢民生保障网，让老区人民的幸福感更具质感、更有温度。

着力优先发展教育事业。优先保障教育投入，进一步调整优化结构，提高教育经费使用效率。继续实施第三期学前教育行动计划。积极推进城乡教育一体化，促进义务教育优质均衡发展。坚持“五统一”，推进智慧学校建设。加快普及高中教育。推进市特殊教育中心迁建、六安一中扩建和义务教育段15所学校等项目建设。支持皖西学院开展地方应用型高水平大学建设，推进职业教育产教融合工程。加强特殊教育，办好继续教育。严格落实新时代教师职业行为十项准则，完善中小学教师待遇保障机制，让广大教师安心从教、热心从教。

着力加强就业和社会保障工作。积极落实各项促进就业政策，统筹推进高校毕业生、农民工等重点群体就业和退役军人安置工作。保障农民工工资按时足额支付，持续增加城乡居民收入。进一步做好退役军人服务管理保障工作。深入推进全民参保计划，健全新型城乡社会救助体系，完善最低生活保障和“三类人群”的住房保障制度。积极应对人口老龄化，深化养老服务体系建设，强化居家养老和社区养老功能。做好留守儿童、困境儿童救助保护和失能特困人员集中养护工作。

着力推进健康六安建设。加快医院现代管理制度建设。加强紧密型医共体建设。强化医疗监管。推进医保支付制度改革，严格控制医疗费用不合理增长。全面推开公立医院编制“周转池”制度，实行基层医务人员“县管乡用”。加快智慧医院建设。大力发展中医药事业。加快市人民医院河西分院、市中医院二期等项目建设。加强疾病预防控制。加快社会办医步伐。做好人口和优生优育工作。

着力繁荣发展文化和体育事业。建成市文化馆、科技馆、图书馆，积极推进市体育中心、市广播电视发射塔等项目建设。大力发展文化产业，积极推进六安恒大童世界及六安文化旅游城建设。引导和鼓励社会力量参与公共文化建设。加强文物保护、开发和利用，推进非遗保护传承。支持引导优秀文艺作品创作生产。深入开展全民健身活动，积极举办、承办重大体育赛事活动。继续打造 “六安国际马拉松”“毅行六安”等文化体育活动品牌。

着力提升平安六安建设水平。按不冷落群众、不推诿群众、不亏待群众、不误导群众的要求，扎实做好信访工作，实现信访形势根本好转。健全社会治安防控体系，深入开展“守护平安”系列行动，推进“雪亮工程”建设，依法打击和惩治黄赌毒等违法犯罪活动，保持扫黑除恶高压态势。坚持“管行业必须管安全、管业务必须管安全、管生产经营必须管安全”，持续开展“铸安”行动，坚决遏制较大及以上生产安全事故。推进大别山天气雷达建设，加强自然灾害防范，提高综合防灾减灾救灾和应急处置能力。强化食品药品安全监管，保障人民群众饮食用药安全。加强新时代全民国防教育、国防动员、人民防空和双拥优抚安置，推进军民融合发展，争创第四届全国双拥模范城，促进军政军民团结。深入推进“七五”普法，扎实做好第四次全国经济普查。维护民族团结、宗教和睦。加强和改进新形势下反邪教工作。

（十）加强政府自身建设，全面提高履职水平。落实年度目标任务，是对政府系统的一场严考。我们要牢固树立“四个意识”，坚持政治建设为统领，坚持以人民为中心的发展思想，驰而不息加强自身建设，全面提高履职水平。

强化法治思维。坚持依法行政，严守宪法和法律，自觉运用法治思维和法治方式推动工作，大力推进政府工作规范化、程序化、法治化。积极推进政务公开，加强政策解读，主动回应公众关切。更加自觉接受人大法律监督、工作监督和政协民主监督，广泛听取民主党派、工商联、无党派人士和人民团体意见，主动接受社会和舆论监督。充分发挥工会、共青团、妇联等人民团体重要作用，着力创建青年发展型城市。完善依法科学民主决策机制，凡涉及公众利益的重大事项，都要深入听取各方意见包括批评意见，让政府的所有工作都体现人民的意愿，让政府工作效果最终由人民来评判。

强化担当作为。加强新理论、新知识学习，全面增强“八种本领”。坚持少说空话、多做实事、扎扎实实、埋头苦干，坚持领导干部 “带着干、教着干、盯着干”，推进工作落实。大兴调查研究之风，重总结经验更重发现问题，做到“临事而惧、好谋而成”。深入贯彻激励新时代新担当新作为的政策措施，完善激励约束、容错纠错机制，旗帜鲜明地为担当干事者鼓劲撑腰，对懒政怠政者严肃问责。建立大督查机制，完善政府工作谋划、布置、实施、督查、反馈、推进、考评机制建设，确保政策措施、目标任务落实到位。

强化正风肃纪。坚定不移落实全面从严治党主体责任和“一岗双责”，持续提升政府系统从严管党治党水平。严格落实中央八项规定精神，坚决整治“四风”问题特别是形式主义、官僚主义，严格规范督查检查考核，严格控制总量和频次。进一步打造“统一规范、公开透明、服务高效、监督到位”的公共资源交易市场。加强对权力运行的制约和监督，严格执行政策跟踪审计和经济责任审计，强化结果应用。支持纪委监委机关依法依规履行职责，建设廉洁政府。

各位代表！

使命重在担当，奋斗铸就辉煌。让我们更加紧密地团结在以习近平同志为核心的党中央周围，在中共六安市委的坚强领导下，锐意进取，奋力开拓，扎实苦干，为全面建成小康社会收官打下决定性基础，开创新时代幸福六安建设新局面，以优异成绩迎接中华人民共和国成立70周年！
